# Selecting the Best Image Pairs to Measure Slope Deformation

**DOI:** 10.3390/s20174721

**Published:** 2020-08-21

**Authors:** Wentao Yang

**Affiliations:** Three-Gorges Reservoir Area (Chongqing) Forest Ecosystem Research Station, School of Soil and Water Conservation, Beijing Forestry University, Beijing 100083, China; yang_wentao@bjfu.edu.cn; Tel.: +86-135-2062-8051

**Keywords:** slope deformation detection, Sentinel-2 images, uncertainty analysis

## Abstract

Optical remote sensing images can be used to monitor slope deformation in mountain regions. Abundant optical sensors onboard various platforms were designed to provide increasingly high spatial–temporal resolution images at low cost; however, finding the best image pairs to derive slope deformation remains difficult. By selecting a location in the east Tibetan Plateau, this work used the co-registration of optically sensed images and correlation (COSI-Corr) method to analyze 402 Sentinel-2 images from August 2015 to February 2020, to quantify temporal patterns of uncertainty in deriving slope deformation. By excluding 66% of the Sentinel-2 images that were contaminated by unfavorable weather, uncertainties were found to fluctuate annually, with the least uncertainty achieved in image pairs of similar dates in different years. Six image pairs with the least uncertainties were selected to derive ground displacement for a moving slope in the study area. Cross-checks among these image pairs showed consistent results, with uncertainties less than 1/10 pixels in length. The findings from this work could help in the selection of the best image pairs to derive reliable slope displacement from large numbers of optical images.

## 1. Introduction

Slope deformations are important precursors for imminent landslide hazards, which pose serious threats to mountain communities around the world [1,2,3]. With increases in population sizes and economic activities, detecting possible slope deformation in mountain regions is becoming increasingly important [4].

Optical remote sensing has been increasingly used to monitor slope deformations [5,6,7,8]. Image correlation is the most frequently used methods to derive slope deformation from optical images [5,6,8,9,10,11]. The principle of deformation detection in optical images is to examine texture similarities with sliding windows in a pair of two images taken on different dates [12]. Thus, the minimum deformation that can be detected depends on the spatial resolution of the optical images. Compared to SAR images, optical remote sensing images are easier to process and are more reliable in the detection of large displacements [13,14,15,16,17,18,19].

There are several factors that can influence the performance of the image correlation methods [11]. Errors in DEM during image orthorectification, misalignments in images, different viewing angles of the remote sensors and illumination differences of the landscape during image acquisitions can result in uncertainties in deriving slope deformations [20]. To derive reliable ground displacement, a stable zone should be selected to compensate for misalignments in image pairs. The mean displacement in the stable zone shows the misalignment in an image pair, and the standard deviation indicates uncertainties from other sources (e.g., different viewing angles of the sensors, illumination differences, etc.).

Quantifying uncertainties in image pairs is crucial for deriving reliable displacements [11]. Lacroix et al. [10] used 22 Sentinel-2 images over nine months, and found that the uncertainties were correlated with temporal intervals between both images in a pair. Yang et al. [8] selected seven Sentinel-2 images from 2015 to 2018, and found that the uncertainties were correlated with solar zenith angles. Bontemps et al. [11] used displacement redundancy from image pairs to reduce uncertainty in the image time series. However, these earlier works used a limited number of image pairs; furthermore, the temporal patterns of uncertainties are still unclear, making it difficult to select the best image pairs among optical images.

This work used the Sentinel-2 images to uncover temporal patterns of uncertainty to derive deformations. Launched by the European Space Agency, the Sentienl-2 mission has two solar-orbiting satellites, 2A and 2B [21]. The Sentinel-2A satellite was launched in June 2015, and the Sentinel-2B satellite was launched in March 2017. Full operation of both satellites could cover anywhere on Earth, with a revisiting time of less than five days. The multi spectral instrument (MSI) sensor on board has a 10-m spatial resolution for near infrared (NIR), with red, green, and blue bands. By far, the Sentinel-2 constellation is the most frequently used public optical Earth observation system.

By using all available Sentinel-2 images in the east Tibetan Plateau, the objectives of this work were (1) to quantify temporal patterns of uncertainties in deriving slope displacement and (2) to test the full capacity of all available Sentinel-2 images, to derive ground deformation information from optical images.

## 2. Materials and Methods

### 2.1. Study Area

**Figure 1:**
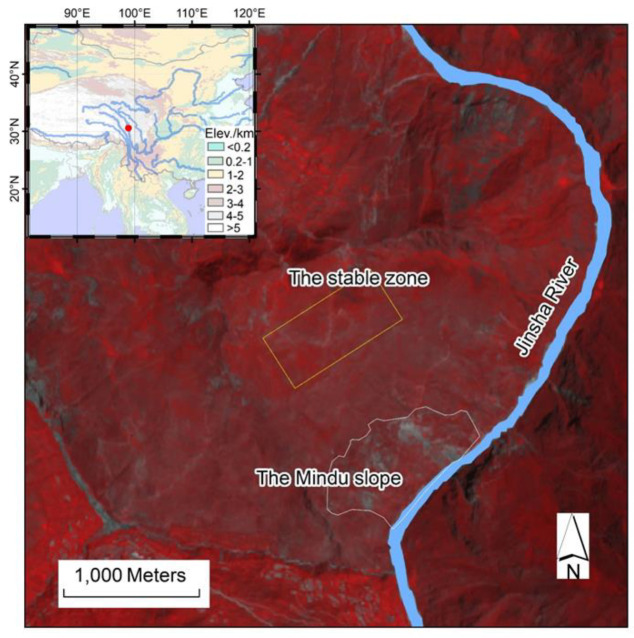
A false color composite Sentinel-2 image acquired on 15 July 2017. The inset map shows the geographical location of the study area.

The study area was located on the eastern Tibetan Plateau along the Jinsha River (Figure 1). The river flows southward, bordering two provincial regions in West China, namely, the Tibet Autonomous Region (AR) and Sichuan Province. The slope of interest was located on the right bank of the Jinsha River, near the Mindu village in the Tibet AR (represented by a white polygon in Figure 1). The elevation of this region ranges from 2600 m to 4000 m. The terrain of this region is characterized by a V-shape valley, indicating severe river incision in the upper Yangtze River on the east margin of the Tibetan Plateau. A series of parallel north-south faults runs through this slope indicated by the Chinese Geological map (1:2,500,000). West and east of these faults are Upper Paleozoic strata and Permian sandstones, respectively. Tensile cracks are visible in high spatial resolution Google Earth images, indicating possible historic slope movement. A monsoon climate dominates the region, with the most precipitation occurring in the summer months, from May to October [22].

To carry out the image correlation analysis, a stable zone was selected to derive possible image misregistration. The stable zone (represented by a yellow quadrangle in Figure 1) in this work was selected on the same slope as the Mindu slope, which is about 600 m apart from the moving slope.

### 2.2. Data

**Figure 2:**
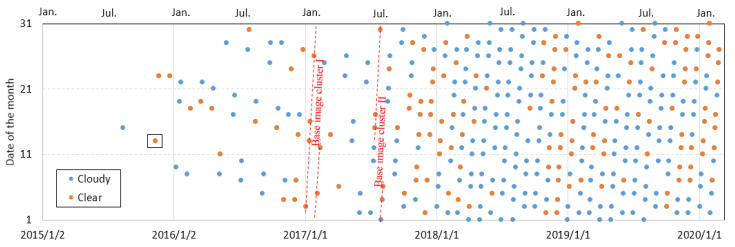
All available Sentinel-2 images covering the Mindu slope until 7 February 2020.

In this study area, 402 Sentinel-2 images were taken until 7 February 2020 (Figure 2). In this work, we only used Sentinel-2 level 1-C product, which is orthorectified before distribution. The earliest Sentinel-2 image was taken on 15 August 2015. Overall, there were 4 Sentinel-2 images taken in 2015, 36 in 2016, 60 in 2017, 140 in 2018, and 142 in 2019. With images defined as cloudy or clear by only examining the Mindu slope and the stable zone, 65.7% (264) of all images were considered to be cloudy and could not be used. Most clear observations were from the winter seasons (from November to February). In this work, only clear images were used (represented by red points in Figure 2).

### 2.3. Time Series of Standard Deviations in E/W and N/S Displacements

The Co-registration of Optically Sensed Images and Correlation (COSI-Corr) method was used to derive ground displacement from the Sentinel-2 images [12]. The method required two optical images taken on different dates, i.e., one acquired earlier as the base image and one acquired later as the target image. Using the frequency of the statistic correlator engine embedded in the COSI-Corr, ground displacement from the target image could be derived against the base image. The frequency correlator engine detects ground displacement by transforming images into Fourier domains, thereby producing better results than the statistic engine. To apply the frequency engine, parameters of two window sizes were predefined, namely, an initial window size and a final window size. The initial window was set no smaller than the final window. An earlier study indicated that larger window sizes led to clearer spatial patterns by depressing background noise, but smaller windows were more sensitive in detecting slope deformation [8]. As the investigation of the window size was not the scope of this work, a medium window size combination of 64–32 was used, which provided merits of both smaller and larger window sizes. Outputs of the COSI-Corr included three map layers, i.e., displacements in the east/west (E/W) and north/south (N/S) directions and a signal-to-noise (SNR) layer.

Uncertainties of the COSI-Corr method were measured using the standard deviations of displacements in a stable zone (represented by the yellow rectangular in Figure 1). The smaller the standard deviation of displacement in the stable zone, the more reliable the image pair was considered to be, regarding displacement derivation.

To derive ground displacement, two images of different dates were used for image correlation analysis, i.e., an earlier base image and a later-acquired target image. For a series of 138 clear images, the first clear image on 13 November 2015 was used as the base, and the other 137 clear images from 2015 to 2020 were used as target images, allowing a time series of standard deviations of displacement to be produced from the stable zone.

### 2.4. Estimated Slope Displacements in 2019 and 2020

**Figure 3:**
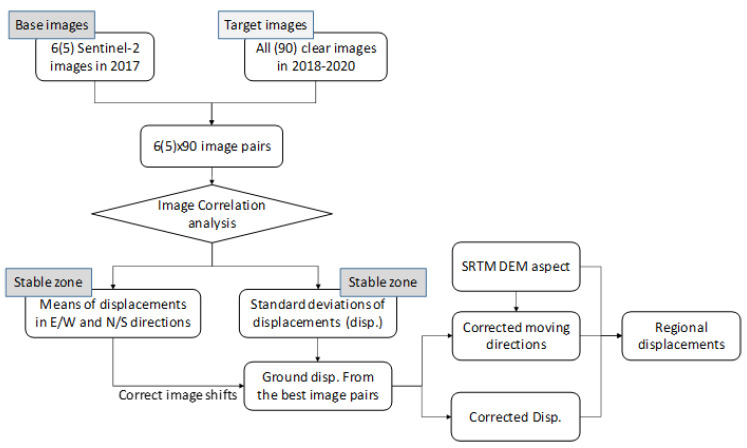
A flowchart to derive the best slope displacement from 2018 to 2020.

A previous study indicated that this Mindu slope was relatively stable (total displacement less than 2 m) from November 2015 to November 2018, and moved (>5 m of displacement) from November 2018 to November 2019 [22]. Therefore, any image in the stable period could be used as the base image to derive accumulated displacement in the later moving periods. In this work, two base image clusters from 2017 (represented by red dots on the red dashed lines in Figure 2) were used to derive ground displacement after January 2018 (Figure 3). For the first base image cluster, six clear images from January and February 2017 were used as base images. For the second base image cluster, five clear images from July and early August 2017 were used as base images to estimate slope displacement. Images within each base cluster were taken on similar dates, and no ground displacement was assumed to occur. The target images for all base images included 90 clear images from January 2018 to 7 February 2020, whereby each base image from 2017 was used to form an image pair with these 90 clear Sentinel-2 images after January 2018. There were 6 × 90 and 5 × 90 image pairs in the first and second cluster pairs, respectively.

For each cluster pair, standard deviations of ground displacement were derived from the stable zone. Image pairs with the least standard deviations in the stable zone were regarded as the most reliable results. Among these best image pairs, the top few that shared the same target image were selected to cross-validate the derived displacements. To derive slope deformation, the mean E/W and N/S displacements from the stable zone were used to correct the image shifts, for pixels with SNR values larger than 0.9. Aspects to constrain displacement were also used to filter out displacement with contradictory directions [22].

## 3. Results

### 3.1. Uncertainty Analysis

#### 3.1.1. Time Series of Standard Deviations in the Stable Zone Using the Base Image from 13 November 2015

**Figure 4:**
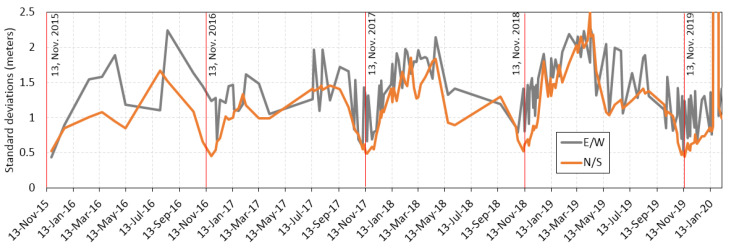
Uncertainties measured as standard deviations in the stable zone for all clear Sentinel-2 images, using the image from 13 November 2015 as the base image.

A clear image from 13 November 2015 was used to compose image pairs with the other clear images, to derive a time series of standard deviations of E/W and N/S displacements in the stable zone. Figure 4 shows the temporal changes of both the E/W and N/S standard deviations. Although there were more sharp changes in the standard deviations of the E/W direction than that of the N/S, uncertainties in the derived slope displacements showed similar temporal patterns of annual fluctuation. The smallest standard deviations occurred in November of each year, which was similar to the date of the base image.

#### 3.1.2. Time Series of Standard Deviations Using the First Base Image Cluster from January and February 2017

**Figure 5:**
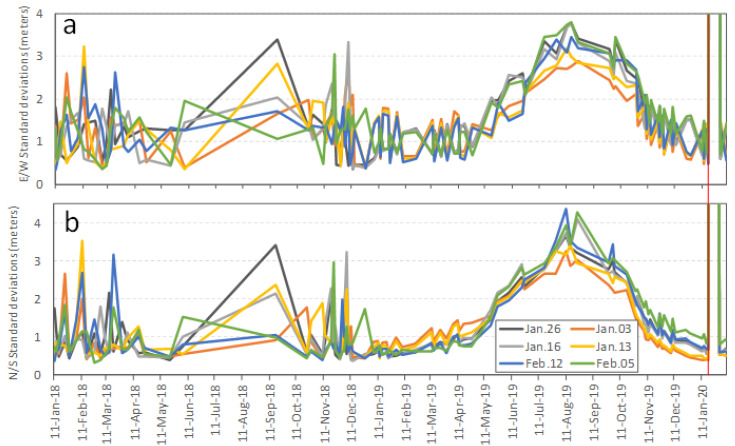
Standard deviations of the derived east/west (E/W) (**a**) and north/south (N/S) displacements (**b**) in the stable zone for 2018–2020, using the six base images taken in January and February 2017.

To further investigate the temporal patterns of uncertainties, six base images from January and February 2017 were used to derive standard deviations in the stable zone, using all of the clear images taken from 2018 to 2020. Figure 5 shows the E/W and N/S standard deviations of ground displacement in the stable zone. Similar to the temporal patterns described by all of the images, as shown in Figure 4, both E/W and N/S uncertainties fluctuated periodically, and the smallest uncertainties in both directions were achieved at the same time of the year as the base images (from January to March). Ubiquitous large E/W and N/S standard deviations occurred in the summer months, from May to October. The standard deviations in the E/W direction changed more dramatically than those of the N/S direction, indicating that the uncertainties in the E/W direction were less predictable than those of the N/S direction.

#### 3.1.3. Time Series of Standard Deviations Using the Second Base Image Cluster from July and August 2017

**Figure 6:**
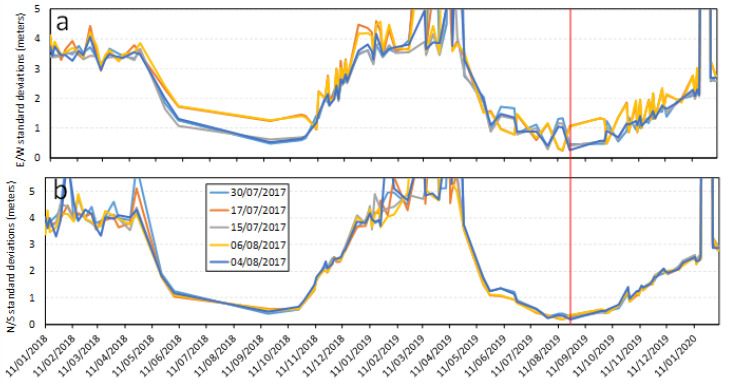
Standard deviations of the derived E/W (**a**) and N/S displacements (**b**) in the stable zone for 2018–2020, using five base images taken in July and August 2017 (second base image cluster).

Another five base images taken in July and August 2017 were used to derive ground displacement from 2018 to 2020. Standard deviations of E/W and N/S displacement in the stable zone are shown in Figure 6. Uncertainties in the stable zone also showed similar temporal variation patterns regarding annual fluctuations, and the smallest uncertainties were shown to be from July to October, i.e., the same season as that of the base images. As seen from these three uncertainty plots in Figure 4, Figure 5 and Figure 6, the minimum standard deviations in displacement of the stable zone were probably achieved using images from the same dates in different years.

### 3.2. Cross-Checks of Derived Displacements

#### 3.2.1. Cross-Checks of the Derived Displacement on 18 January 2020

**Figure 7:**
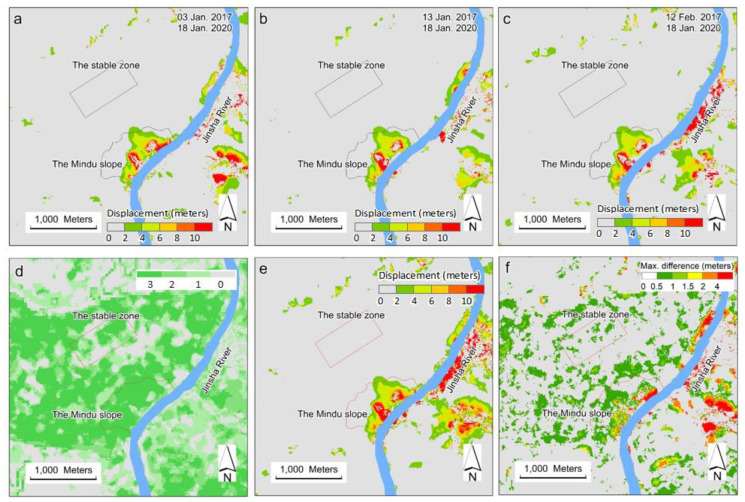
Derived slope displacement from 18 January 2020 using three base images taken on 3 January (**a**), 13 January (**b**), and 12 February 2017 (**c**), respectively. Valid results in (**a**–**c**) were counted per pixel and shown in (**d**). Means and ranges of displacement from (**a**–**c**) were shown in (**e**,**f**), respectively.

A previous study showed that the slope was stable (total displacement of less than 2 m) from November 2015 to November 2018, and moved from November 2018 [22]. In theory, there should be no difference in using any base image in this stable period to derive slope displacement from a given target image during the moving period. In this work, displacement was derived on 18 January 2020 using six base images in January and February 2017. These derived results would theoretically be the same and could be used to cross-validate each other. Although there were six displacement results for each target image, only three with the least E/W standard deviations were selected. The base images for these three displacements were images taken on 3 January, 13 January, and 12 February 2017, respectively. Their derived displacements are shown in Figure 7a–c. Based on these three results, the valid results (0–3 in Figure 7d) and their means and ranges of displacement (Figure 7e–f) were determined for each pixel. Figure 7a–c show that the spatial patterns of the three derived results on the Mindu slope were quite consistent. All of the results indicated more displacement near river the bank and less on the upper slope. Some pixels on the east of the Mindu slope near the river showed large ranges (>2 m), whereas the ranges of results in most parts of the slope were within 1 m. Because all of these images were acquired during the winter season and the solar angles were low, some background noise was present in the results unrelated to the moving slope, probably caused by mountain shadows. Despite the use of some of the best image pairs within the time series, some differences regarding the same target image still exist (Figure 7f).

#### 3.2.2. Cross-Checks of Derived Displacement from 24 August 2019

**Figure 8:**
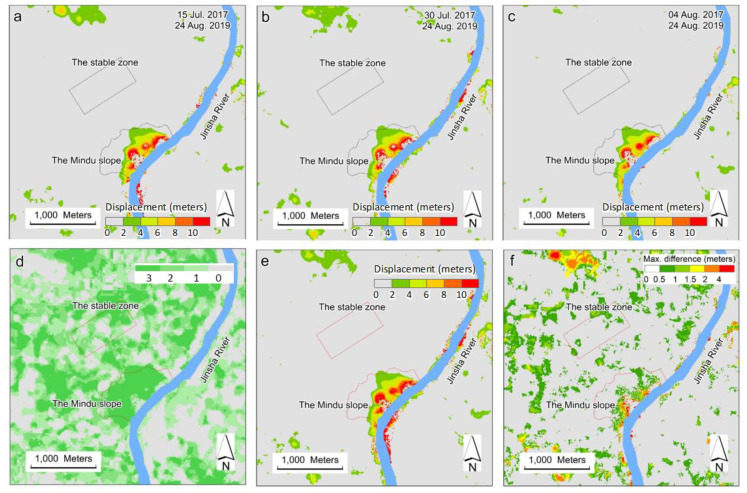
Derived slope displacement from 24 August 2019, using three base images taken on 15 (**a**), 30 (**b**) July and 4 August (**c**) 2017, respectively. Valid results in (**a**–**c**) were counted per pixel and shown in (**d**). Means and ranges of displacement from (**a**–**c**) were shown in (**e**,**f**), respectively.

Three results from 24 August 2019 with the least standard deviations are shown in Figure 8. The base images for these three results were taken on 15 July, 20 July, and 4 August 2017. Figure 8a–c show that the spatial patterns of the derived ground displacements are very alike. Because all base and target images shown in Figure 8 are from summer months (either July or August), the solar angles are large and there are few mountain shadows seen in the images, which may explain the lack of background noise in Figure 8 compared to Figure 7. Results from 24 August 2019 also showed greater displacement near the riverbanks than the upper slopes, which was spatially consistent with the images shown in Figure 7.

#### 3.2.3. Comparison of Displacement between August 2019 and January 2020

**Figure 9:**
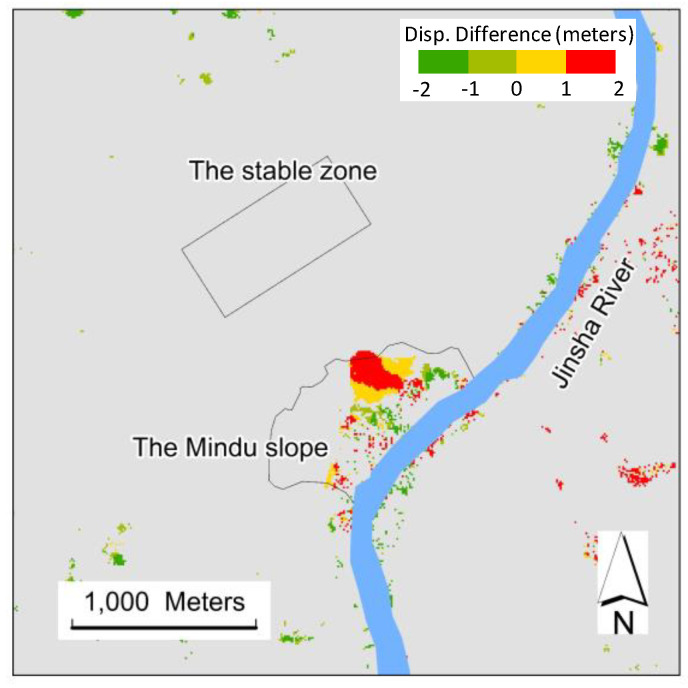
Difference in derived ground displacement between January 2020 and August 2019.

To further check the derived slope deformation, the derived displacements from January 2020 (in Figure 7e) and August 2019 (Figure 8e) were compared. The difference in mean displacement between these two periods was calculated using pixels with mean displacement values larger than 2 m and ranges less than 1 m. Figure 9 shows that the most detected displacement changes were positive from August 2019 to January 2020, showing a good spatial pattern and probably demonstrating the slipperiness of the slope.

## 4. Discussion

### 4.1. Temporal Patterns of Uncertainties in Slope Deformation Detection Using Optical Images

Previous works show that uncertainties in deriving slope deformation from optical images could result from DEM errors during image orthorectification, different viewing angles of the sensors and illumination differences of the landscape during image acquisitions [20]. Yang et al. [8] hypothesize that using similar dates spanning different years could lead to lower uncertainties. However, we know little about the temporal patterns of uncertainties, as few studies used long-term observations to study temporal patterns of uncertainties. By using multiyear dense optical images, this work showed that uncertainties fluctuate annually, and the least uncertainty can be achieved by using images of the same dates in different years. This finding makes it easier to select the best image pairs from a large number of images to detect reliable slope displacement.

Using more image pairs may not lead to better results in slope deformation detection [22]. On the contrary, including many low-quality results could dwarf reliable results [11]. By selecting image pairs with the least uncertainty, the most reliable results could be easily singled out and used preferentially.

Sentinel-2 is a European Space Agency’s mission to monitor earth surface dynamics, which has two twine satellites in orbit, and can revisit anywhere on earth between 56° south and 84° north every 5 days at the equator [21]. In this work, we used all available Sentinel-2 images to monitor ground displacement of a slope in the east Tibetan Plateau. At full capacity in 2019–2020, there were intervals of one or two days between every two observations for this study area, which enabled the most frequent observations with a spatial resolution of 10 m [21]. Although the Sentinel-2 demonstrated very frequent observations in this study area, it suffered from constant cloud contamination, due to the study area having a monsoon climate, with most of the precipitation occurring in the summer months, from May to October [22]. Our work with Sentinel-2 time series data indicate that local climate could seriously affect the efficiency of using optical images. In addition, unfavorable weather conditions also pose a problem for InSAR techniques when deriving slope deformation [23].

### 4.2. Limitations

Standard deviations of displacement in the stable zone are widely regarded as a reliable measure of uncertainty [8,10,11], with the logic being that displacement within a stable zone should exhibit the same values in all pixels and any inconsistency regarded as uncertainty in a pair. However, derived ground displacements and uncertainties can be influenced by window sizes. As mentioned by Yang et al. [8], larger window sizes lead to smooth results by suppressing background noise, which could also lead to more homogeneous displacement patterns and low standard deviations in the stable zone. However, standard deviations in the stable zone may not represent true uncertainties of ground measurements. Therefore, using a large window size to decrease standard deviation may not necessarily reduce uncertainty when deriving displacement. Future works should focus on true measurements to validate these results.

## 5. Conclusions

In this work, all available Sentinel-2 images on 7 February 2020 in a mountain region in the east Tibetan Plateau were used. We found the Sentinel-2 images had a very short revisiting time of two/three days in this study area, but unfavorable weather conditions contaminated 65.7% of all observations. Using the remaining clear Sentinel-2 images, an image correlation method was applied to assess the temporal patterns of uncertainty in deriving slope deformation. We found, for a given base image, displacement uncertainties fluctuated annually, and the smallest uncertainty can be achieved by using target images taken on similar dates. As more optical images are becoming available to the public at low costs, selecting the best ones from a large number of optical images to derive slope displacements is becoming more difficult. This uncovered temporal pattern of uncertainty can facilitate the selection of optimal images to derive reliable ground displacements.
